# Predicting COVID-19 Symptoms From Free Text in Medical Records Using Artificial Intelligence: Feasibility Study

**DOI:** 10.2196/37771

**Published:** 2022-04-27

**Authors:** Josefien Van Olmen, Jens Van Nooten, Hilde Philips, Annet Sollie, Walter Daelemans

**Affiliations:** 1 Department of Family Medicine and Population Health University of Antwerp Antwerp Belgium; 2 Computational Linguistics, Psycholinguistics and Sociolinguistics Research Centre University of Antwerp Antwerp Belgium

**Keywords:** natural language processing, text mining, electronic medical records, COVID-19, structured registry, coding procedure, prediction model, feasibility study, precision model, artificial intelligence, primary care

## Abstract

**Background:**

Electronic medical records have opened opportunities to analyze clinical practice at large scale. Structured registries and coding procedures such as the International Classification of Primary Care further improved these procedures. However, a large part of the information about the state of patient and the doctors’ observations is still entered in free text fields. The main function of those fields is to report the doctor’s line of thought, to remind oneself and his or her colleagues on follow-up actions, and to be accountable for clinical decisions. These fields contain rich information that can be complementary to that in coded fields, and until now, they have been hardly used for analysis.

**Objective:**

This study aims to develop a prediction model to convert the free text information on COVID-19–related symptoms from out of hours care electronic medical records into usable symptom-based data that can be analyzed at large scale.

**Methods:**

The design was a feasibility study in which we examined the content of the raw data, steps and methods for modelling, as well as the precision and accuracy of the models. A data prediction model for 27 preidentified COVID-19–relevant symptoms was developed for a data set derived from the database of primary-care out-of-hours consultations in Flanders. A multiclass, multilabel categorization classifier was developed. We tested two approaches, which were (1) a classical machine learning–based text categorization approach, Binary Relevance, and (2) a deep neural network learning approach with BERTje, including a domain-adapted version. Ethical approval was acquired through the Institutional Review Board of the Institute of Tropical Medicine and the ethics committee of the University Hospital of Antwerpen (ref 20/50/693).

**Results:**

The sample set comprised 3957 fields. After cleaning, 2313 could be used for the experiments. Of the 2313 fields, 85% (n=1966) were used to train the model, and 15% (n=347) for testing. The normal BERTje model performed the best on the data. It reached a weighted F1 score of 0.70 and an exact match ratio or accuracy score of 0.38, indicating the instances for which the model has identified all correct codes. The other models achieved respectable results as well, ranging from 0.59 to 0.70 weighted F1. The Binary Relevance method performed the best on the data without a frequency threshold. As for the individual codes, the domain-adapted version of BERTje performs better on several of the less common objective codes, while BERTje reaches higher F1 scores for the least common labels especially, and for most other codes in general.

**Conclusions:**

The artificial intelligence model BERTje can reliably predict COVID-19–related information from medical records using text mining from the free text fields generated in primary care settings. This feasibility study invites researchers to examine further possibilities to use primary care routine data.

## Introduction

Electronic medical records (EMRs) have opened the opportunity to analyze clinical practice at large scale, and to perform clinical-epidemiological research, which can inform health care managers and policy makers. Structured registries and coding procedures such as the International Classification of Primary Care have improved the way doctors put information into EMR, which has facilitated the use of its output and accelerated research using these data. The free text fields also still available in EMR systems have been hardly used apart from clinical follow-up. Yet the usage of this information has great potential to contribute to monitoring and evaluation of clinical practice and to EMR-driven research. In 2016, US researchers compared the accuracy for case detection of diagnoses such as dementia, stroke, diabetes, and depression based upon coded information versus the procedure including free text, and they found a significant improvement in algorithm sensitivity in the latter [1].

This is not surprising since these fields contain the core of clinical practice captured in the encounter notes. The encounter notes available in most EMRs have a structured “SOAP” format, which stands for Subjective (patient’s history), Objective (physical examination), Assessment (initial differential diagnosis), and Plan [2]. The main function of these free text fields is to report the doctor’s line of thought, to remind oneself and colleagues on follow-up actions, and to be accountable for clinical decisions. Therefore, they contain the richest data about the state of the patient and the observations of the doctor. Yet their use is also challenging. Health care providers tend to write notes quickly, with personal styles and abbreviations, and they vary in their completeness and quality of reporting. Therefore, encounter notes have seldom been used for further analyses and research.

A 2019 review on the use of free text fields in the EMR [3] showed that the focus of most studies was on the development of methods to extract symptom information for disease classification tasks. For instance, a UK study validated a method for mining free text fields to link them to frequent medical conditions such as colic or renal failure [4]. The analysis of symptoms themselves has been restricted to specific and rather narrow domains such as neuromuscular diseases [5], psychiatry [6], and veterinary medicine [7,8]. A recent study demonstrates the feasibility of extracting information from free text notes and using this as input to a model for predicting patient outcomes [9].

To use the information from free text fields at a large scale, methods to recognize this information need to be developed and evaluated. A 2012 study found that combination of a manually created filter and rule learning algorithm yielded the best performance across two different data sets (radiology reports and general practitioner [GP] notes) [10], but the performance for the GP set was considerably lower. The variation of symptoms and note-taking is peculiar for the GP domain. This implies that more such studies are necessary to develop robust methods for data recognition for GP data sets to improve the reproducibility of data and their value for routine use.

The relevance for quick information using real time data was apparent in the COVID-19 pandemic. The collection, evaluation, and synthesis of information started quickly. Data mainly came from hospital settings, where most severe cases were admitted, and where resources could be mobilized quickly, for instance, to make decision-support algorithms for diagnosis and treatment based upon models that predict disease outcomes [11]. This predominant use of data from severely ill patients led to risk of bias in the models [12]. This underlined the need to develop methods to extract data quickly and reliably from primary care health records at large scale.

Our study contributes to this goal. The objective of this paper was to develop a robust method to transform the primary care notes into a list of symptoms that could feed improved COVID-19 prediction models through the development of a text classifier model that can predict the relevant symptoms (output) based upon the analysis of the free text fields (input). If this method proves robust, free text data from primary care clinical notes about COVID-19–related symptoms can be mined at large scale quickly and reliably.

## Methods

### Background

This study is part of the project ID-CoV to develop procedures for data identification, harmonization, and linkage to develop robust methodologies to build a risk prediction tool based on primary care and hospital data for the identification of individuals at higher risk for severe COVID-19 outcomes (project id 43639, Funded by University of Antwerp).

### Data Collection

The iCAREdata database was used, which is a database of contacts in out of hours (OOH) care by general practice cooperatives, triage centers (additional centers organized during the COVID-19 pandemic to triage between infectious and noninfectious diseases), pharmacies, and a small number of first aid departments connected to the system (covering OOH care of roughly two-thirds of Flanders population) [13]. One OOH hosts between 80 and 150 different GPs. Data from EMR at OOH services therefore cover a broad range of different physicians, with different approaches of medical care and registration of clinical data, leading to high variability of content, completeness, quality, and format of information in the data set, which adds methodological challenges to developing mining procedures. Nevertheless, the analysis of the data of this segment of primary care consultations is especially relevant in a pandemic context [14]*.* The units of analysis in iCAREdata are records, each record being one contact (=consultation). Due to the exploratory nature, sample size was not considered a limiting factor. We aimed to use as many observations (patient’s encounters) as possible in a given time period to reduce the uncertainty of our model estimates. A study database was created that comprises all records from January 1, 2019, to November 30, 2020. These are roughly 779,000 records, which include a pre–COVID-19 period and a COVID-19 epidemic period (March 1, 2020, to November 30, 2020).

For each record, 15 fields were extracted (Multimedia Appendix 1). For the data mining study reported in this paper, only 5 fields were used (Textbox 1). The “field subjective” (physician’s report on the patient’s account of their problem) and “field objective” (findings and measurements of the physician) were explored for relevant text (combinations). We used supervised machine learning algorithms to classify information into one or more of predetermined symptoms via the multiclass, multilabel prediction model described below. Fields “DiagnTekst” and “DiagnCod” were used as control records for validation.

The establishment of the symptom list that needed to be the outcome of the classifier model was started from an initial list of 23 symptoms identified by the Belgium Public Health Institute as relevant [15] but was refined driven by the data. A manual exploration of the data set yielded 62 symptoms most of them with a negative counterpart, indicating the absence of that symptom. Negative symptoms were relevant because of their negative predictive value in a diagnostic or prognostic algorithm [16]; for instance, the absence of cough contributing to the likelihood or non-likelihood of a COVID-19 diagnosis. The skewed distribution led to a regrouping of symptoms, resulting in a final list of 27 signs or symptoms (Table 1). There are two types of symptom codes, which are “objective,” based on the “objectief” text field, and “subjective,” based on the “subjectief” text field, respectively.

**Relevant fields for input to machine learning algorithm to recognize signs and symptoms.**

Machine learning fields

- IdContact: unique id for contact (date, guard post, time)
- Subjectief: subjective text field
- Objectief: objective text field
- DiagnTekst: diagnosis term (thesaurus)
- DiagnCod: diagnosis code from the International Classification of Primary Care [17]

**Table 1:** Final list with signs and symptoms to be coded from the free text.

Final symptoms—coded	Explanation
S^a^1; SA^b^1	Cough
S100; SA100	Upper respiratory tract infection complaints
S101; SA101	Dyspnea and shortness of breath
S7; SA7	Thoracic pain or chest pain
S102; SA102	Loss of taste or smell
S10; SA10	History of fever
S112	Pain or stiffness in muscles, joints, or neck
S109	Complaints of throat or voice
S12	Fatigue
S15	Headache
S103; SA103	Gastrointestinal complaints
S104	Significant acute event or change
S105	Chronic pulmonary complaints; smoking; potentially worsening
S105	Other comorbidities or being pregnant
S106	Known cardiovascular diseases or hypertension or relevant medication
S107	Known diabetes or diabetes medication
S108	Medication NSAID^c^ or immunosuppressive drugs
S113	Palpitations or dizziness
S110	General complaints as malaise and illness
S111	Mental or sleeping problems
S63	Close contact with a sick person (COVID-19 symptoms) or COVID-19–positive case
O^d^101	Respiratory signs found during physical examination
O6	Fever measured by health care staff
O102	Ear-, nose-, or throat-positive signs during physical examination
O104	Neurological symptoms
O103	Circulatory positive signs: abnormal pulse rate, tension, or turgor of capillary refill
O19	Impression of being ill

^a^S: Subjective.

^b^A: absence of the symptom.

^c^NSAID: nonsteroidal anti-inflammatory drugs.

^d^O: Objective.

### Development of a Classifier Model

Classification entails the tasks of predicting the class (or label of output variable—the list with 27 signs or symptoms) based upon the input variables (the free text fields). Two approaches were examined to develop a multiclass, multilabel categorization classifier, which are as follows: (1) a classical machine learning–based text categorization approach; and (2) a deep neural network learning approach based on fine-tuning a pretrained model for domain adaptation and learning the classification task. The advantage of the latter approach is that, in general, less supervised training data (ie, annotated data) are needed for learning the task. A random sample from the data set was extracted for annotation, with a distribution of 1/3 records from before the start of the COVID-19 pandemic (operationalized as March 1, 2020) and 2/3 after that date, comprising 3957 entries in total. Character encoding problems in the text data were solved during preprocessing. Empty entries and entries that did not contain any information (eg, “/”) in either the subjective or objective fields were removed from the data set, which left 2313 entries to be used for the experiments. The subjective and objective text fields were merged into one text field in order to receive sufficiently large text fragments for prediction. The same resulting text could be assigned multiple objective and subjective codes. Negative symptoms were kept apart by coding them with an A-label; for instance, SA10 indicated the absence of a history of fever. The A codes were frequent among the objective text fields. Entries that were annotated as irrelevant (without any symptom code) were used as negative examples for training of the models.

The samples were annotated by 5 medical doctors or researchers. Inter-annotator variability was checked. All annotators started annotation of the same set and manually compared inconsistencies, discussed them, adapted the standard operating guidelines, and repeated this procedure until agreement of 90% was achieved. During the annotation phase, the inventory of symptom tags (classes) evolved, but all annotated data were made comparable through a common code book and standard operating procedure in the final data set. The number of entries, average number of tokens (instances of words and punctuation marks), and total amount of tokens for the training partition, test partition, and the total data set are summarized in Table 2.

**Figure 1:**
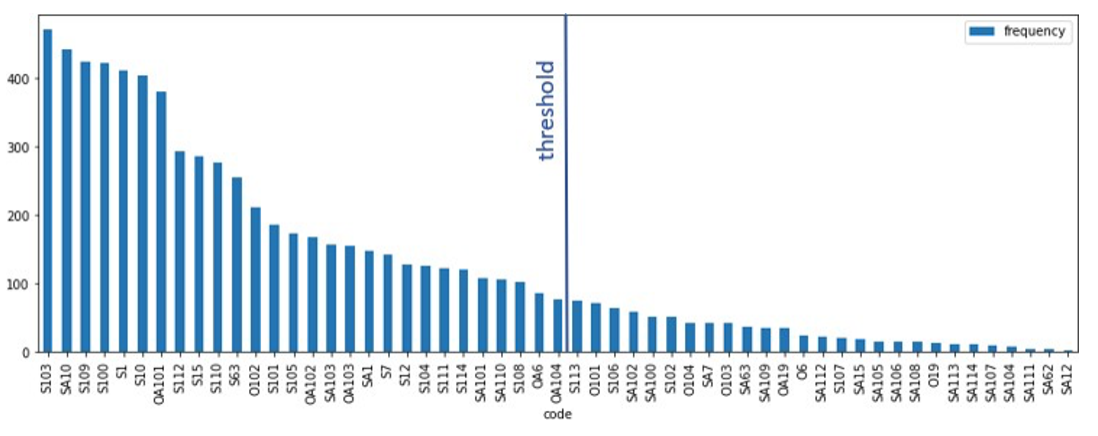
Code distribution in the data set. Codes to the right of the threshold line were removed for the experiments where a frequency threshold was employed.

**Figure 2:**
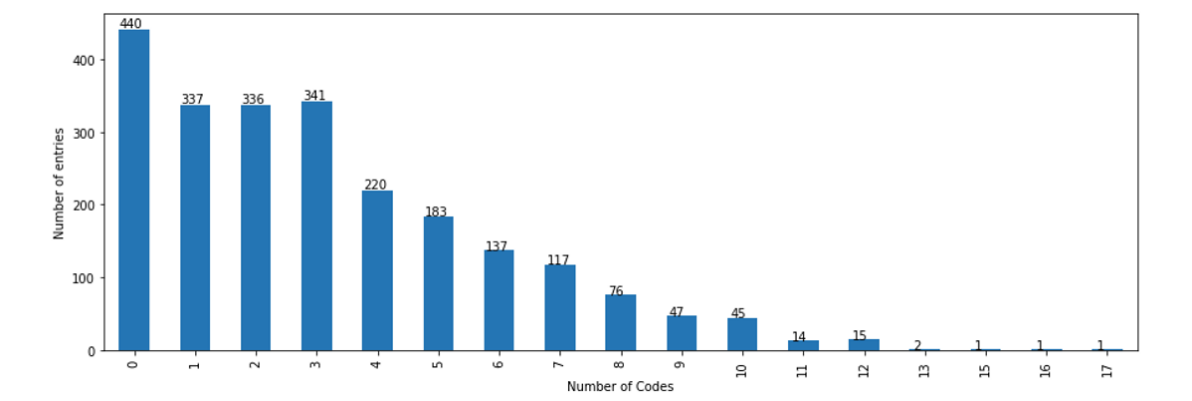
Distribution of the percentage of entries in the data set assigned to a particular number of codes.

The distribution of codes (labels) in the data set is shown in Figures 1 and 2. The majority of the codes are subjective codes; out of the 55 codes, 43 (78%) are subjective while the remaining 12 (22%) are objective. For the development of the classifier, experiments were conducted with all codes and only codes occurring at least 50 times, which meant 35 (63%) out of 55 codes (representing 93% of all used codes).

**Table 2:** Total number of entries, average amount of tokens per entry, and total amount of tokens for the training, test portions, and the entire data set.

Portion	Entries, n (%)	Average tokens per entry, n	Total tokens, n
Train	1966 (85)	24	53,929
Test	347 (15)	31	10,779
Total	2313 (100)	28	64,708

The baseline accuracies (most frequent class prediction and random prediction) are 0.15 and 0.08, respectively. In the first set of experiments, we used classic machine learning methods. One of the most common approaches to multiclass, multilabel classification is Binary Relevance. With this method, the multilabel problem is translated to *n* binary classification problems, where *n* is equal to the number of labels present in the data set. Binary in this case means that the classifier attempts to predict whether a class (code) is present (1) or not (0) in the text. For the binary classifiers, we used the Stochastic Gradient Descent classifier [18] and optimized the hyperparameters (including the loss function) by performing a gridsearch on them (a search for the best combination of algorithm parameters on a validation partition of the training data in the context of 5-fold cross-validation). The performance of this method is measured by taking the mean of all cross-validated results from the individual binary classifiers.

Further experiments were then conducted with BERTje [19], a Dutch version of BERT [20]. BERT is a widely used model for natural language processing, and the availability of a Dutch version BERTje made it the first choice of the team. BERTje is an open-source pretrained language model that has been trained on a large amount of generic (nonmedical) Dutch text data. Thus, the model already has knowledge about language patterns before having been trained on data for a specific problem, in contrast to, for example, the Stochastic Gradient Descent classifier, which was limited to the training data. Additionally, we continued the pretraining of BERTje by using a selection of the text fields of the original data set (part of the iCAREdata database) in order to “adapt” BERTje to medical texts. This method has been proven to be successful on a wide range of tasks [21,22]. For all experiments, the F1 macro score metric was used for evaluation, which is the average F1 score (harmonic mean of precision and recall) obtained for the classes. In our binary relevance setup and the implementation of F1 macro we used, only successful predictions of the minority class (correctly predicting that the code is present) are taken into account, which makes it the most critical (but also the most relevant) evaluation.

For all experiments, we used a stratified train-test split, where 80% of the data were used for training and hyperparameter optimization, and 20% were used for testing. The best model on test (BERTje) was then fine-tuned on all annotated data and applied to the complete (unannotated) data set, predicting diagnostic codes based on the text fields.

### Ethics Approval

Ethical approval was acquired through the Institutional Review Board of the Institute of Tropical Medicine and the ethics committee of the University Hospital of Antwerpen (ref 20/50/693).

## Results

In the tables below, the results of the experiments on the test set are summarized. Across all models that were trained and tested on data with a frequency threshold for the labels, the normal BERTje model performed the best on the data, reaching a weighted F1 score of 0.70 and an exact match ratio or accuracy score of 0.38 (Table 3), indicating the instances for which the model has identified all correct codes. The results per code can be found in Table S1 of Multimedia Appendix 1. The other models achieved respectable results as well, ranging from 0.59 to 0.70 weighted F1. The Binary Relevance method performed the best on the data without a frequency threshold (Table S2 of Multimedia Appendix 1).

Regarding the results on the individual codes themselves, the domain-adapted version of BERTje performs better on several of the less common objective codes (O101, O102, OA101, OA102, OA104, and OA6), while BERTje reaches higher F1 scores for the least common labels (S102 and SA102) especially, and most other codes in general.

**Table 3:** Average results for the different models on test data with a frequency threshold for the codes (codes occurring at least 50 times).

Method	Weighted precision	Weighted specificity	Weighted recall	Weighted F1
Binary Relevance (SGD^a^ classifier)	0.69	0.93	0.52	0.59
BERTje	0.77	0.97	0.68	0.70
BERTje (domain adaptation)	0.74	0.96	0.62	0.67

^a^SGD: Stochastic Gradient Descent.

## Discussion

### Principal Findings

In this paper, we demonstrated the feasibility of developing a model to predict symptom codes from primary care clinical text notes. Across the three models tested, the pretrained neural network model BERTje performed the best. The reason for the lower performance of the domain-adapted BERTje needs further investigation. Neural networks can forget information they previously learned upon learning new information (catastrophic forgetting); however, from the current data, we are not able to explain if this was the reason for the lower performance.

Our model resulted in the ability to predict symptoms from the free text with a weighted average F score of 0.66 (0.75 sensitivity and 0.97 specificity) on all codes, regardless of frequency, and an F score of 0.70 (0.77 sensitivity and 0.97 specificity) on codes that occurred more than 50 times in the data set. Very few studies that have developed mining techniques for clinical notes, in general [23], and from primary care, in particular. Yet the incidental other studies show feasibility and good results [24]. A study using a Repeated Incremental Pruning to Produce Error Reduction rule learning model resulted in a sensitivity of 0.91, and a specificity 0.76 [10]. To our knowledge, this is the first study that mined data from OOH health care organizations.

The strength of our study is that we used a large database representative of a population of 6 million people in Flanders and with many different GPs. The major limitation of our study relates to the quality of the raw data. The data set contained consultations of OOH primary care consultations. The notes in these consultations were often very brief, and the completeness and quality of information varied across entries. This is similar in studies from routine primary care [25]; however, in OOH care, this is likely to be worse, making it more difficult to develop mining models. This reflects the reality of medical practice and the limitations of real-world data. Further research into minimal needs for reporting for both clinical and other purposes is warranted. Another limitation is that some symptom codes, for instance SA100 (*geen BLWI klachten-no respiratory tract complaints)* could not be learned by the machine learning models. The explanation for this, as for similar cases, is that there were too few instances available in the data set for the model to learn from [9]. For these codes, it would be useful to investigate the data for more cases to be annotated. Even if more elaborate annotating will improve the gain, not all free text fields can be transformed into coded information, which needs to be taken into account in the interpretation of the output.

Notwithstanding the limitations, our study is relevant for primary care research and evaluation. Once coded, these symptoms can be monitored, evaluated, and processed, for the development and testing of algorithms, for near real time symptom surveillance [26], or for assessing quality of history taking and record keeping. Our study focused on symptom detection, but wider applications of the text mining and natural language processing can be thought of, such as the analyses of adverse events or patient-reported experiences [23].

### Conclusions

The BERTje prediction models can reliably predicting COVID-19–related information from medical records using text mining from the free text fields generated in primary care settings. The feasibility to convert this rich but largely untapped source of clinical encounter into data usable for monitoring, evaluation, and research provides opportunities for comprehensive analysis of primary care consultations at large scale, as well as use for monitoring purposes, also in other primary care settings. This feasibility study invites researchers to examine further possibilities to use primary care routine data, for instance, to examine the process of clinical reasoning through EMR analysis or to assess the input of patient-related information into the diagnostic process.

## Appendix

Multimedia Appendix 1 Details about experiments.

## Abbreviations

EMR: electronic medical record
GP: general practitioner
OOH: Out of Hours
