# Supplementary material for: Predicting COVID-19 Symptoms From Free Text in Medical Records Using Artificial Intelligence: Feasibility Study
Source: JMIR Med Inform. 2022 Apr 27;10(4):e37771. doi: 10.2196/37771 (PMC9049643; doi:10.2196/37771)
Supplement: Multimedia Appendix 1 [file medinform_v10i4e37771_app1.docx]

Multimedia Appendix 1

Details about experiments.

## Software Details

All code for the experiments was written in Python 3.7.6.

For the development of the classical machine learning algorithm, sci-kit learn [25] (version 1.0.2) was used for the implementation of the SGDClassifier and score metrics. For the development of the BERT-based models, we used the implementation of the models available on Huggingface^[[1]](#footnote-1)^. To use these models, we used the transformers library (version 4.11.0) and PyTorch^[[2]](#footnote-2)^ (version 1.9.0).

## Additional Results

In Table S1, the results for each code with a frequency threshold are summarized in terms of precision, recall, f1, sensitivity and specificity. Table 6 summarizes the same results for all codes in the dataset.

*Table S1: Results per code (with frequency threshold) of the best model in terms of precision, recall, f1-score (performance on positive class), sensitivity and specificity.*

|  | **precision**  **(sensitivity)** | **recall** | **f1-score** | **specificity** | **frequency in test** |
| --- | --- | --- | --- | --- | --- |
| **O101** | 0.5 | 0.15 | 0.24 | 0.99 | 13 |
| **O102** | 0.82 | 0.77 | 0.79 | 0.98 | 35 |
| **OA101** | 0.83 | 0.88 | 0.85 | 0.96 | 59 |
| **OA102** | 0.78 | 0.56 | 0.65 | 0.99 | 25 |
| **OA103** | 0.5 | 0.42 | 0.46 | 0.97 | 26 |
| **OA104** | 0.4 | 0.13 | 0.2 | 0.99 | 15 |
| **OA6** | 1.0 | 0.2 | 0.33 | 1.0 | 15 |
| **S1** | 0.92 | 0.95 | 0.94 | 0.98 | 62 |
| **S10** | 0.84 | 0.92 | 0.88 | 0.96 | 61 |
| **S100** | 0.87 | 0.86 | 0.86 | 0.97 | 63 |
| **S101** | 0.92 | 0.8 | 0.86 | 0.99 | 30 |
| **S102** | 1.0 | 0.5 | 0.67 | 1.0 | 8 |
| **S103** | 0.81 | 0.77 | 0.79 | 0.95 | 70 |
| **S104** | 0.57 | 0.21 | 0.31 | 0.99 | 19 |
| **S105** | 0.33 | 0.61 | 0.43 | 0.89 | 28 |
| **S106** | 0.25 | 0.08 | 0.12 | 0.99 | 13 |
| **S108** | 0.33 | 0.05 | 0.09 | 0.99 | 19 |
| **S109** | 0.92 | 0.94 | 0.93 | 0.98 | 63 |
| **S110** | 0.69 | 0.52 | 0.59 | 0.96 | 46 |
| **S111** | 0.56 | 0.43 | 0.49 | 0.98 | 21 |
| **S112** | 0.76 | 0.74 | 0.75 | 0.97 | 43 |
| **S113** | 0.73 | 0.57 | 0.64 | 0.99 | 14 |
| **S114** | 0.54 | 0.41 | 0.47 | 0.98 | 17 |
| **S12** | 0.53 | 0.39 | 0.45 | 0.98 | 23 |
| **S15** | 0.98 | 0.95 | 0.96 | 1.0 | 43 |
| **S63** | 0.94 | 0.76 | 0.84 | 0.99 | 38 |
| **S7** | 0.83 | 0.58 | 0.68 | 0.99 | 26 |
| **SA1** | 0.88 | 0.68 | 0.77 | 0.99 | 22 |
| **SA10** | 0.87 | 0.79 | 0.83 | 0.97 | 66 |
| **SA100** | 0.0 | 0.0 | 0.0 | 1.0 | 9 |
| **SA101** | 0.79 | 0.58 | 0.67 | 0.99 | 19 |
| **SA102** | 0.5 | 0.67 | 0.57 | 0.99 | 3 |
| **SA103** | 0.55 | 0.48 | 0.51 | 0.97 | 23 |
| **SA110** | 1.0 | 0.12 | 0.22 | 1.0 | 16 |
| **micro avg** | 0.78 | 0.68 | 0.72 | 0.98 | 1053 |
| **macro avg** | 0.7 | 0.54 | 0.58 | 0.97 | 1053 |
| **weighted avg** | 0.77 | 0.68 | 0.7 | 0.97 | 1053 |

*Table S2: Results per class of the best model in terms of precision, recall, f1-score (performance on positive class), sensitivity and specificity for all codes, irrespective of the frequency threshold.*

|  | **precision** | **recall** | **f1-score** | **specificity** | **support** |
| --- | --- | --- | --- | --- | --- |
| **O101** | 1.0 | 0.08 | 0.15 | 1.0 | 12 |
| **O102** | 0.77 | 0.77 | 0.77 | 0.97 | 35 |
| **O103** | 0.25 | 0.14 | 0.18 | 0.99 | 7 |
| **O104** | 1.0 | 0.14 | 0.25 | 1.0 | 7 |
| **O19** | 0.0 | 0.0 | 0.0 | 1.0 | 4 |
| **O6** | 0.0 | 0.0 | 0.0 | 1.0 | 6 |
| **OA101** | 0.81 | 0.92 | 0.86 | 0.95 | 59 |
| **OA102** | 0.77 | 0.4 | 0.53 | 0.99 | 25 |
| **OA103** | 0.48 | 0.4 | 0.43 | 0.97 | 25 |
| **OA104** | 0.5 | 0.2 | 0.29 | 0.99 | 15 |
| **OA19** | 0.0 | 0.0 | 0.0 | 1.0 | 5 |
| **OA6** | 1.0 | 0.07 | 0.13 | 1.0 | 14 |
| **S1** | 0.94 | 0.95 | 0.94 | 0.99 | 61 |
| **S10** | 0.83 | 0.9 | 0.87 | 0.96 | 61 |
| **S100** | 0.93 | 0.84 | 0.88 | 0.99 | 63 |
| **S101** | 0.91 | 0.7 | 0.79 | 0.99 | 30 |
| **S102** | 1.0 | 0.62 | 0.77 | 1.0 | 8 |
| **S103** | 0.83 | 0.7 | 0.76 | 0.96 | 70 |
| **S104** | 0.67 | 0.11 | 0.18 | 1.0 | 19 |
| **S105** | 0.58 | 0.42 | 0.49 | 0.98 | 26 |
| **S106** | 0.5 | 0.08 | 0.14 | 1.0 | 12 |
| **S107** | 0.0 | 0.0 | 0.0 | 1.0 | 3 |
| **S108** | 0.5 | 0.11 | 0.17 | 0.99 | 19 |
| **S109** | 0.9 | 0.9 | 0.9 | 0.98 | 63 |
| **S110** | 0.57 | 0.48 | 0.52 | 0.95 | 44 |
| **S111** | 0.5 | 0.22 | 0.31 | 0.99 | 18 |
| **S112** | 0.86 | 0.7 | 0.78 | 0.98 | 44 |
| **S113** | 0.67 | 0.31 | 0.42 | 0.99 | 13 |
| **S114** | 0.5 | 0.35 | 0.41 | 0.98 | 17 |
| **S12** | 0.59 | 0.43 | 0.5 | 0.98 | 23 |
| **S15** | 0.98 | 0.93 | 0.95 | 1.0 | 43 |
| **S63** | 0.91 | 0.79 | 0.85 | 0.99 | 38 |
| **S7** | 0.9 | 0.75 | 0.82 | 0.99 | 24 |
| **SA1** | 0.76 | 0.59 | 0.67 | 0.99 | 22 |
| **SA10** | 0.88 | 0.79 | 0.83 | 0.98 | 66 |
| **SA100** | 0.0 | 0.0 | 0.0 | 1.0 | 9 |
| **SA101** | 0.89 | 0.44 | 0.59 | 1.0 | 18 |
| **SA102** | 0.5 | 0.33 | 0.4 | 1.0 | 3 |
| **SA103** | 0.65 | 0.59 | 0.62 | 0.98 | 22 |
| **SA104** | 0.0 | 0.0 | 0.0 | 1.0 | 2 |
| **SA105** | 0.0 | 0.0 | 0.0 | 1.0 | 2 |
| **SA106** | 0.0 | 0.0 | 0.0 | 1.0 | 4 |
| **SA107** | 0.0 | 0.0 | 0.0 | 1.0 | 2 |
| **SA108** | 0.0 | 0.0 | 0.0 | 1.0 | 2 |
| **SA109** | 0.0 | 0.0 | 0.0 | 1.0 | 3 |
| **SA110** | 0.56 | 0.29 | 0.38 | 0.99 | 17 |
| **SA111** | 0.0 | 0.0 | 0.0 | 1.0 | 0 |
| **SA112** | 0.0 | 0.0 | 0.0 | 1.0 | 0 |
| **SA113** | 0.0 | 0.0 | 0.0 | 1.0 | 3 |
| **SA114** | 0.0 | 0.0 | 0.0 | 1.0 | 2 |
| **SA12** | 0.0 | 0.0 | 0.0 | 1.0 | 0 |
| **SA15** | 0.0 | 0.0 | 0.0 | 1.0 | 1 |
| **SA62** | 0.0 | 0.0 | 0.0 | 1.0 | 2 |
| **SA63** | 0.0 | 0.0 | 0.0 | 1.0 | 4 |
| **SA7** | 0.0 | 0.0 | 0.0 | 0.99 | 3 |
| **micro avg** | 0.8 | 0.62 | 0.7 | 0.99 | 1100 |
| **macro avg** | 0.47 | 0.32 | 0.36 | 0.98 | 1100 |
| **weighted avg** | 0.75 | 0.62 | 0.66 | 0.97 | 1100 |

*Table S3: Results per class of the best model in terms of precision, recall, f1-score (performance on positive class), sensitivity and specificity for all codes, using the binary relevance method, irrespective of the frequency threshold.*

|  | **precision** | **recall** | **f1-score** | **specificity** | **support** |
| --- | --- | --- | --- | --- | --- |
| **SA102** | 0.65 | 0.58 | 0.61 | 0.99 | 57 |
| **OA103** | 0.53 | 0.42 | 0.47 | 0.95 | 170 |
| **S103** | 0.8 | 0.55 | 0.65 | 0.89 | 474 |
| **OA102** | 0.66 | 0.44 | 0.53 | 0.96 | 162 |
| **SA12** | 0 | 0 | 0 | 1 | 2 |
| **S105** | 0.38 | 0.19 | 0.26 | 0.93 | 181 |
| **S104** | 0.21 | 0.23 | 0.22 | 0.95 | 138 |
| **S113** | 0.64 | 0.29 | 0.4 | 0.97 | 85 |
| **SA7** | 0.78 | 0.41 | 0.54 | 0.99 | 44 |
| **S114** | 0.38 | 0.3 | 0.33 | 0.96 | 132 |
| **S109** | 0.89 | 0.73 | 0.8 | 0.94 | 454 |
| **S108** | 0.66 | 0.48 | 0.56 | 0.97 | 108 |
| **SA106** | 0 | 0 | 0 | 0.99 | 17 |
| **SA1** | 0.77 | 0.55 | 0.64 | 0.97 | 157 |
| **SA111** | 0 | 0 | 0 | 1 | 5 |
| **S100** | 0.74 | 0.62 | 0.68 | 0.91 | 458 |
| **S1** | 0.82 | 0.75 | 0.78 | 0.94 | 435 |
| **O6** | 0.06 | 0.04 | 0.04 | 0.99 | 27 |
| **SA104** | 0 | 0 | 0 | 1 | 4 |
| **SA107** | 0 | 0 | 0 | 1 | 11 |
| **SA105** | 0 | 0 | 0 | 0.99 | 16 |
| **S15** | 0.85 | 0.74 | 0.8 | 0.96 | 298 |
| **O19** | 0 | 0 | 0 | 0.99 | 17 |
| **OA104** | 0.4 | 0.19 | 0.26 | 0.97 | 89 |
| **SA108** | 0 | 0 | 0 | 0.99 | 17 |
| **S101** | 0.56 | 0.59 | 0.57 | 0.96 | 194 |
| **SA63** | 0.29 | 0.25 | 0.27 | 0.99 | 40 |
| **SA110** | 0.18 | 0.22 | 0.2 | 0.96 | 118 |
| **OA19** | 0.18 | 0.09 | 0.12 | 0.99 | 33 |
| **S7** | 0.68 | 0.51 | 0.58 | 0.97 | 144 |
| **SA62** | 0 | 0 | 0 | 1 | 4 |
| **SA10** | 0.83 | 0.64 | 0.72 | 0.92 | 459 |
| **S102** | 0.81 | 0.37 | 0.51 | 0.98 | 57 |
| **S111** | 0.41 | 0.29 | 0.34 | 0.96 | 136 |
| **SA112** | 0.92 | 0.55 | 0.69 | 1 | 22 |
| **S10** | 0.8 | 0.58 | 0.67 | 0.91 | 426 |
| **S63** | 0.76 | 0.58 | 0.65 | 0.94 | 285 |
| **SA100** | 0.33 | 0.04 | 0.07 | 0.98 | 53 |
| **S12** | 0.49 | 0.36 | 0.42 | 0.96 | 125 |
| **O103** | 0.22 | 0.24 | 0.23 | 0.99 | 42 |
| **S106** | 0.14 | 0.16 | 0.15 | 0.97 | 69 |
| **S107** | 0.89 | 0.4 | 0.55 | 0.99 | 20 |
| **SA101** | 0.67 | 0.43 | 0.52 | 0.97 | 115 |
| **SA113** | 0 | 0 | 0 | 0.99 | 15 |
| **S110** | 0.39 | 0.4 | 0.4 | 0.91 | 298 |
| **SA103** | 0.63 | 0.45 | 0.52 | 0.96 | 161 |
| **OA101** | 0.75 | 0.7 | 0.73 | 0.94 | 389 |
| **SA114** | 0.33 | 0.14 | 0.2 | 0.99 | 14 |
| **SA109** | 0.62 | 0.44 | 0.52 | 0.99 | 34 |
| **OA6** | 0.23 | 0.2 | 0.21 | 0.97 | 79 |
| **S112** | 0.75 | 0.56 | 0.64 | 0.94 | 283 |
| **O101** | 0.4 | 0.33 | 0.36 | 0.98 | 69 |
| **O102** | 0.7 | 0.43 | 0.54 | 0.94 | 220 |
| **SA15** | 0.9 | 0.5 | 0.64 | 1 | 18 |
| **O104** | 0.05 | 0.04 | 0.05 | 0.98 | 48 |
| **micro avg** | 0.65 | 0.51 | 0.57 | 0.98 | 7528 |
| **macro avg** | 0.46 | 0.33 | 0.37 | 0.97 | 7528 |
| **weighted avg** | 0.65 | 0.51 | 0.57 | 0.94 | 7528 |

1. https://huggingface.co/models [↑](#footnote-ref-1)
2. https://pytorch.org/ [↑](#footnote-ref-2)
